# ReFerm® a Fermented Oat Gruel Composition, Improves Colonic Barrier Function and Modulates Tricellulin Expression in Patients With Irritable Bowel Syndrome

**DOI:** 10.1111/nmo.70381

**Published:** 2026-06-23

**Authors:** Vittorio Abruzzese, Olga Biskou, Martin E. Winberg, Hans Israelsen, Olga Bednarska, Susanna Walter, Åsa V. Keita

**Affiliations:** ^1^ Department of Biomedical and Clinical Sciences Linköping University Linköping Sweden; ^2^ Nordic Rebalance A/S Hillerød Denmark; ^3^ Department of Gastroenterology Linköping University Hospital Linköping Sweden; ^4^ Department of Health, Medicine, and Caring Sciences Linköping University Linköping Sweden

**Keywords:** fermented oats, functional gastrointestinal disorder, gut permeability, intestinal barrier, irritable bowel syndrome, tight junctions

## Abstract

**Background:**

Intestinal barrier dysfunction has been implicated in the pathophysiology of irritable bowel syndrome (IBS), contributing to increased permeability, low‐grade inflammation, and symptom generation. Microbial compositions are known to improve epithelial barrier function, although the underlying molecular mechanisms remain elusive. The aim was to investigate the effects of the fermented oat gruel preparation ReFerm® on colonic barrier function and tight junction protein expressions in patients with IBS.

**Methods:**

Colonic biopsies from patients with IBS were mounted in Ussing chambers to assess the direct effects of ReFerm® on epithelial permeability. In parallel, biopsies from patients treated with ReFerm® or placebo by enema were analyzed for the expression of multiple tight junction proteins using Western blotting and confocal microscopy. In vitro effects were further evaluated in Caco‐2 cells.

**Key Results:**

ReFerm® reduced both paracellular and transcellular permeability when added directly to biopsies mounted in Ussing chambers, compared with unstimulated biopsies. This effect was accompanied by increased expression of the tight junction protein tricellulin at tricellular junctions. In vitro experiments in Caco‐2 cells supported these findings, demonstrating direct effects of ReFerm® on epithelial barrier properties.

**Conclusions and Interferences:**

ReFerm® strengthens colonic barrier integrity in the gut mucosa of patients with IBS, potentially through modulation of the tight junction protein tricellulin. These findings provide mechanistic insight into the barrier‐protective effects of fermented oat interventions such as ReFerm® and support the potential therapeutic role in IBS.

## Key Points

- Patients with irritable bowel syndrome (IBS) have been shown to have impaired gut barrier function, which may allow substances to pass more easily through the gut lining and contribute to gut symptoms.
- In this study, a fermented oat‐based preparation, ReFerm®, was shown to reduce the passage of substances through the gut lining and strengthen the gut barrier in colonic biopsies from patients with IBS.
- The barrier‐strengthening effect was linked to increased levels of tricellulin, a protein that helps seal the spaces between gut cells, suggesting a potential mechanism behind the beneficial effects.

## Plain Language Summary

Patients with irritable bowel syndrome (IBS) have an impaired gut barrier function, which may allow substances to pass more easily through the gut lining and contribute to symptoms. We investigated whether a fermented oat‐based preparation, ReFerm®, can strengthen the gut barrier in patients with IBS using two complementary approaches. In one approach, gut biopsies from patients with IBS were exposed to ReFerm® to examine its immediate effects on gut permeability. In parallel, biopsies from patients treated with ReFerm® or placebo by enema were analyzed to assess changes in tight junction proteins that help seal the spaces between the intestinal cells. We found that ReFerm® improves gut barrier function by reducing permeability and increasing levels of the tight junction protein tricellulin, important for maintaining the gut barrier. These findings suggest that fermented oat preparations such as ReFerm® may help protect the gut lining and could have therapeutic potential for patients with IBS.

## Graphical abstract

Patients with irritable bowel syndrome (IBS) have been shown to have impaired gut barrier function, which may contribute to gut symptoms by allowing substances to pass more easily through the intestinal lining. In this study, the fermented oat‐based preparation ReFerm® reduced gut permeability and strengthened the gut barrier in colonic biopsies from patients with IBS, an effect associated with increased levels of the tight junction protein tricellulin.

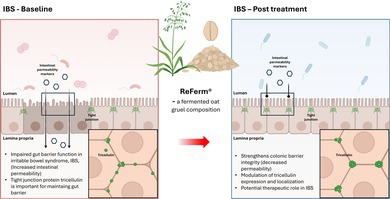

## 1 Introduction

Irritable Bowel Syndrome (IBS) is a disorder of the brain‐gut axis associated with abdominal pain and disturbed bowel habits [1], in which impaired intestinal barrier function has emerged as a key pathophysiological feature [2, 3, 4]. Stress, food allergens, infections, and dysbiosis of the gut microflora are recognized triggers, capable of increasing intestinal permeability, thereby sustaining a state of low‐grade inflammation and visceral hypersensitivity [5]. A range of pharmacological, dietary, and psychological interventions are used in the management of IBS, reflecting the multifactorial nature of the disorder, although several approaches are supported by limited clinical evidence [6]. A better understanding of key pathophysiological mechanisms, including intestinal barrier dysfunction, may help improve targeted treatment strategies.

The intestinal mucosa consists of a single layer of epithelial cells connected by tight junctions (TJ), which are essential for maintaining epithelial integrity and regulating paracellular permeability [7, 8]. These junctions are primarily formed by claudins, whose organization into sealing strands determines barrier properties; some claudins, such as claudin‐1, are barrier‐forming, whereas others confer increased paracellular permeability. TJ proteins are linked to the cytoskeleton via scaffolding proteins such as ZO‐1 [9, 10] supporting junctional structure and function. In addition to claudins, the TJ‐associated MARVEL protein family contributes to barrier regulation, including occludin and tricellulin [11, 12]. Occludin is predominately localized at bicellular junctions (bTJ) and is involved in the regulation of barrier permeability [12], whereas tricellulin is specifically enriched at tricellular junctions (tTJ) [11], where three epithelial cells meet. At these sites, tricellulin is essential for sealing the central tub‐like structure formed by the TJ network, thereby playing a key role in maintaining epithelial barrier integrity. In IBS patients, a reduced expression of occludin and ZO‐1 has been demonstrated compared with healthy controls (HC), and the subcellular distribution of multiple TJ proteins appears disrupted, indicating functional alterations rather than changes at the transcriptional level [3, 4]. Bertiaux‐Vandaële et al. [4] have demonstrated that the lower levels of occludin and claudin‐1 observed in patients with IBS compared to HC correlate with longer symptom duration and greater abdominal pain intensity, supporting the concept that barrier dysfunction contributes to visceral hypersensitivity and the generation of IBS symptoms. Interestingly, a recent study by Awad et al. [13] showed a reduction of occludin expression which caused a displacement of tricellulin localization from its normal tricellular junction localization in colonic biopsies of patients with IBS‐M compared with HC.

Studies have shown that TJ protein expressions can be modulated by microbial compositions through multiple mechanisms [14, 15, 16]. ReFerm® is a dietary product manufactured by fermenting an oat composition using *Lactobacillus plantarum* DSM 9843 (synonymous with 299v) [17]. Clinical studies investigating *L. plantarum* DSM 9843 in IBS have reported inconsistent results, with some clinical trials demonstrating symptom improvement [18, 19], while others have shown no significant effect [20]. These discrepancies highlight the need for further studies to better understand the underlying mechanisms and to clarify the therapeutic potential of such interventions. Clinical and experimental studies indicate that probiotic administration is associated with improved epithelial integrity, reflected by increased transepithelial electrical resistance (TER), and reduced markers of bacterial translocation and systemic inflammation, such as IL‐6 and TNF [21]. We previously showed [22] that rectal administration of ReFerm® improved barrier function in patients with IBS as evidenced by decreased paracellular permeability and increased TER compared to placebo. More recently, we showed decreased mast cell degranulation by ReFerm® [23], however, the underlying molecular mechanisms, particularly at the level of TJ proteins, remained elusive. Therefore, in the present study, we investigated the direct effects of ReFerm® on epithelial barrier function by direct application to colonic biopsies from IBS patients mounted in Ussing chambers. Moreover, the expression of multiple TJ proteins was quantitatively analyzed using Western blotting and confocal microscopy in colonic biopsies from IBS patients who self‐administered ReFerm® or placebo as an enema [22]. In addition, the effects of ReFerm® on TJ expressions were assessed in vitro using Caco‐2 cells.

## 2 Material and Methods

### 2.1 Patients

**FIGURE 1:**
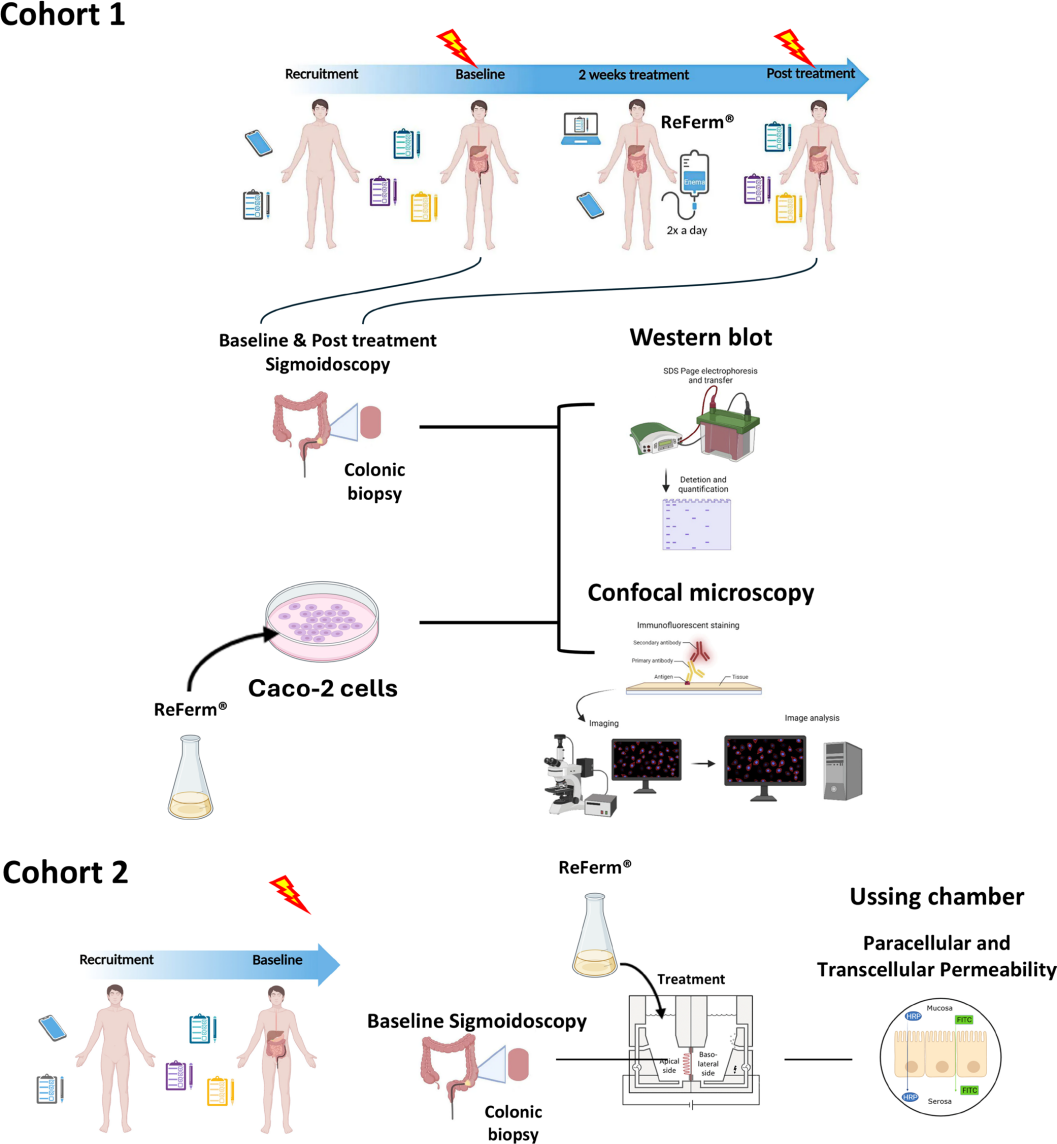
Study design. Two cohorts of patients with moderate‐to‐severe irritable bowel syndrome (IBS) were recruited. Cohort 1 patients were randomized to receive either ReFerm® or placebo. They completed symptom questionnaires, and distal colon biopsies were obtained by sigmoidoscopy at baseline and after 14 days of twice‐daily ReFerm® or placebo enemas. Biopsies were lysed for Western blot analysis of tight junction (TJ) proteins, and additional biopsies were paraffin‐embedded, sectioned, stained for TJ, and examined by confocal microscopy. Caco‐2 cells were treated with ReFerm® or vehicle for Western blotting or fixed for staining and imaging. Colonic biopsies from patients in Cohort 2 were mounted in Ussing chambers and exposed to ReFerm® or vehicle on the mucosal side to assess direct effects on intestinal permeability.

All patients were recruited at the Department of Gastroenterology, University Hospital, Linköping, between December 2020 and November 2023, according to predefined inclusion and exclusion criteria (Table 1), and were divided into two cohorts. The study design is illustrated in Figure 1.

**TABLE 1:** Inclusion and exclusion criteria for patients with IBS and changes in questionnaire outcomes before and after the two interventions.

Inclusion criteria	Exclusion criteria
Age 18–70 years Confirmed IBS‐diarrhea or IBS‐mixed bowel habits according to Rome IV criteria. Moderate to severe IBS according to IBS‐SSS score (≥ 175p) Proficiency in both written and spoken Swedish	Organic gastrointestinal disease History of major gastrointestinal operation, excluding appendectomy and cholecystectomy Psychiatric disease (bipolar disease, schizophrenia) NSAID intake within 14 days before the endoscopy Self‐reported pregnancy

Questionnaire	ReFerm®	Placebo
IBS‐SSS	ns	*z—*2.6, *p* < 0.05
GSRS‐IBS	ns	ns
VSI	*z—*2.69, *p* < 0.05	ns
HADS	ns	ns
SHS	*z—*2.73, *p* < 0.05	*z—*2.19, *p* < 0.05
DWQ	ns	ns

*Note:* Numbers for changes in questionnaire outcomes are presented as z‐scores (standard scores indicating how many standard deviations a data point lies above or below the mean) and *p*‐values. ns, non‐significant.

Abbreviations: DWQ, Daily Web Questionnaire; GSRS‐IBS, Gastrointestinal Symptom Rating Scale–IBS; HADS, Hospital Anxiety and Depression Scale; IBS‐SSS, Irritable bowel syndrome severity scoring system; NSAID, non‐steroidal anti‐inflammatory drugs; SHS, Short Health Scale; VSI, Visceral Sensitivity Index.

Cohort 1 consists of 30 patients (5 men) who fulfilled the Rome IV criteria [24]. The patients had experienced IBS for an average of 13 years (range 2–40 years), the mean age was 37 years (range 19–55 years), and the average body mass index (BMI) was 26 (range 18–41). Based on predominant bowel habits and according to the Rome IV criteria, participants were classified as diarrhea‐predominant (IBS‐D) (*n* = 8) or mixed bowel habits (IBS‐M) (*n* = 22) [25]. All patients had moderate to severe IBS, with a mean symptom severity score of 332.5 (range 180–488) as measured by the IBS severity scoring system (IBS‐SSS). They were randomly assigned to receive ReFerm® (18 patients, two men) or placebo (12 patients, three men). There were no significant differences in age, BMI, or disease severity between the patients receiving ReFerm® and those receiving placebo. Four patients (no men) in the ReFerm® group and two patients (one man) in the placebo group withdrew from the study. Firstly, a single‐blind, randomized experimental trial was performed. Eligible individuals were identified through telephone screening, and since each participant served as their own control, self‐reported allergy was not considered an exclusion criterion, provided that allergic exposure remained unchanged throughout both interventions. Participants were subsequently randomized into one of two groups: ReFerm® or placebo consisting of commercially available thickened water (Thick‐It, Kent Precision Foods Group Inc., Muscatine, IA, USA) [23]. Sigmoidoscopy was conducted at baseline and after 14 days of treatment with either ReFerm® or placebo enemas administered twice daily. The enemas were delivered rectally while the participant was positioned on the left side and were retained for as long as tolerated, but minimum for 10 min (min). Retention was maintained in both the left lateral and supine positions to facilitate retrograde peristalsis. Clinical symptom improvement was assessed using questionnaires administered before and after the intervention period, with participants additionally completing daily questionnaires throughout the 14‐day treatment phase, as described in the questionnaire section. To enhance adherence to the study protocol, a principal investigator (OBe) conducted follow‐up telephone calls twice weekly during the intervention period.

Cohort 2 was specifically included to assess acute direct effects of ReFerm® on epithelial permeability ex vivo, independent of repeated enema exposure. Cohort 2 consists of 8 patients (3 men) who fulfilled the Rome IV criteria. The patients had experienced IBS for an average of 16 years (range 3–35 years); the mean age was 33 years (range 19–50 years), and the average BMI was 25 (range 17–30). Based on predominant bowel habits and according to the Rome IV criteria, participants were classified as IBS‐D (*n* = 2) or IBS‐M (*n* = 6). All patients had moderate to severe IBS, with a mean IBS‐SSS of 355 (range 195–446).

The study is registered as a clinical trial; [https://clinicaltrials.gov/], identifier [NCT05475314].

The study was approved by the Committee of Human Ethics, (Dnr 2020–03485 and 2019–02932) and all participants provided written informed consent.

### 2.2 Sigmoidoscopy and Collection of Biopsies and Plasma

A flexible sigmoidoscopy was performed following standard bowel preparation with an enema, with the endoscope advanced approximately 30–40 cm proximal to the linea dentata. Each participant in Cohort 1 underwent the procedure twice, once at baseline and again after the 14‐day enema intervention with either ReFerm® or placebo. Patients of Cohort 2 underwent the procedure once, at baseline. Biopsies from the distal colon were collected using forceps without a central lance, immediately transferred into ice‐cold oxygenated Krebs buffer (115 mM NaCl, 1.25 mM CaCl_2_, 1.2 mM MgCl_2_, 2 mM KH_2_PO₄, 25 mM NaHCO₃; pH 7.35), and transported to the laboratory. Biopsies were allocated for three different applications. For immediate Ussing chamber experiments, biopsies were kept in Krebs buffer. Additional biopsies were fixed in 4% paraformaldehyde (Histolab, Sweden) in phosphate buffer saline (PBS) for 24 h at 4°C, followed by storage in 70% ethanol prior to paraffin embedding and sectioning at 5 μm. Remaining biopsies were snap‐frozen at −80°C for subsequent Western blot analysis.

Fasting venous blood samples were collected in EDTA‐treated tubes and a mixture of 1.3 mg EDTA and 50 μL Trasylol 10,000 KiE was added to each ml of blood. After centrifugation at 3400 g for 15 min in 4°C, plasma was redrawn and frozen in −80°C until analyzed for TNF and vasoactive intestinal polypeptide (VIP) by enzyme‐linked immunosorbent assay (ELISA) and enzyme immunoassay (EIA), respectively.

### 2.3 The Intervention Product ReFerm®

ReFerm® is a fermented oat‐based preparation containing microbial metabolites, including short‐chain fatty acids such as lactic acid, acetate, and propionate, which are likely contributors to its barrier‐modulating effects. Both the intervention and placebo used in this study are identical to those described in our previous publications [22, 23]. ReFerm® was manufactured using a process consistent with previous methods [17, 22], and to match the viscosity of the intervention product, a commercially available thickened water product (Thick‐it) served as placebo [22]. Full proprietary composition of Thick‐it is not publicly disclosed by the manufacturer; however, Thick‐It does not contain fermentable substrates or bioactive microbial metabolites comparable to ReFerm®.

The same patient material was used, and all handling, processing, and quality‐control procedures followed the previously established protocols without any modifications. For in vitro experiments, a heat‐treated version of ReFerm® was used, as previously described [22]. For more details on the intervention products, authors refer to [22].

### 2.4 Questionnaires

We employed a comprehensive set of validated questionnaires to assess IBS symptoms, psychological factors, and subjective health, following the methodology described in our previous publication [22]. All questionnaires were completed twice, before and after the intervention, while a short web‐based questionnaire was administered daily during the 14‐day intervention period. IBS symptom severity was measured using the IBS‐SSS, and specific gastrointestinal symptom clusters were captured using the Gastrointestinal Symptom Rating Scale‐IBS. Gastrointestinal symptom‐related anxiety was assessed with the Visceral Sensitivity Index, and coexisting anxiety and depression were evaluated using the Hospital Anxiety and Depression Scale. Subjective health status was measured with the Short Health Scale. In addition, daily fluctuations in symptom burden and emotional wellbeing were recorded using an in‐house, non‐validated electronic end‐of‐day questionnaire [22]. For more details on the questionnaires, authors refer to [22].

### 2.5 Ex Vivo Barrier Function

Colonic biopsies were mounted in Ussing chambers. After 30 min of equilibration, 250 μM of the paracellular probe fluorescein isothiocyanate (FITC)‐dextran 4 kDa (Sigma‐Aldrich, USA) and 10 μM of the 44 kDa transcellular probe horseradish peroxidase (HRP) (type VI; Sigma‐Aldrich) were added to the mucosal sides. Additionally, ReFerm® was added to the mucosal side to a final concentration of 1:20 or 1:100. Samples from the serosal sides were collected at 0, 30, 60, 90, and 120 min. Biopsies added Krebs buffer only served as controls (vehicle). The transepithelial potential difference (PD), the TER, and short‐circuit current (Isc) across the biopsies were monitored throughout the experiments to ensure tissue viability.

To determine passage of FITC‐dextran, samples were plated on a black 96‐well plate, as previously described [26]. The intensities of the samples and known standards were measured at 488 nm using a VICTOR X3 multileader plate reader. The optical density of the known standards was used to create a standard curve that was used to determine the concentrations of unknown samples. Passage of HRP was measured using the QuantaBluTM Fluorogenic Peroxidase Substrate Kit (Pierce, Thermo Fisher Scientific, USA), as previously described [26] and passage was further determined using VICTOR X3.

### 2.6 In Vitro Barrier Function

Caco‐2 cells were grown in high‐glucose Dulbecco's modified Eagle's medium (DMEM, Gibco, USA) supplemented with 10% v/v fetal bovine serum (FBS, Gibco, USA), 1% L‐glutamine (Gibco), and 1000 units/mL or μg/mL of penicillin/streptomycin (Gibco), respectively, at 37°C in a 5% CO_2_ incubator. For Western blotting, the cells were plated at a density of 1 × 10^6^ cells per well on 6 well‐plates, while for microscopy, cells were plated on coverslips at a density of 100,000 cells per well on 12 well‐plates. Prior to each experiment, cells were allowed to grow for 14–16 days until a confluent, differentiated monolayer was reached. Every two to three days, the cells were washed with PBS to remove cell debris and dead cells, and the media was replaced. On the day of the experiment, cells were washed with PBS, and the media was replaced with cell culture media supplemented with ReFerm® (diluted 1:20 or 1:100). The cells were incubated with complete culture media containing ReFerm® for up to 24 h. Wells containing only cell culture medium were used as vehicle controls. Individual time points were assessed and optimized in separate wells. All experiments were performed in triplicate and repeated on four independent occasions.

Following incubation with ReFerm®, cells for Western blotting were washed with PBS to remove excess protein. Fresh PBS was added to each well, after which cells were detached using a cell scraper, collected into tubes, and pelleted at 4°C and 300 × g for 5 min. The supernatants were discarded, and the cell pellets were stored at −80°C until analysis.

For microscopy, cell monolayers grown on coverslips were rinsed with PBS and fixed in cold 4% neutral buffered formaldehyde for 10 min, then stored at 4°C until further analysis.

### 2.7 Western Blotting

Protein homogenates from biopsies and Caco‐2 cells were extracted using RIPA buffer (Pierce) supplemented with Mini Protease inhibitor Cocktail (Roche, Germany), 1 mM PMSF and 50 U/mL nuclease (Pierce), as previously described [27]. Briefly, biopsies were homogenized using a Tissue‐Lyser II (Qiagen, Sweden) at 30 frequency/s for 1 min followed by sonication (4 × 5 s at 40% amplitude (Fisherbrand, Fisher Scientific, USA)), end‐over‐end rotation for 30 min at 4°C, and centrifuged at 14000 × g for 30 min at 4°C. Caco‐2 cells were processed identically, except that homogenization was omitted. All samples were boiled in sample buffer for 10 min, and 20 μg of protein per sample was separated on a 4%–12% Tris‐glycine SDS‐gradient gel (Invitrogen, USA). Proteins were transferred onto nitrocellulose membranes (Amersham, Germany), blocked with 5% skim milk for 1 h at room temperature (RT) and incubated overnight at 4°C with primary antibodies against Claudin‐1 (mouse; Invitrogen), Occludin (mouse; Abcam, UK), Tricellulin (rabbit; Invitrogen), ZO‐1 (mouse or goat; Invitrogen or Abcam), β‐actin (mouse or rabbit; Cell Signaling, Invitrogen). Membranes were washed (5 × 5 min) and incubated for 1 h at RT with secondary antibodies (donkey anti‐rabbit 790 and donkey anti‐mouse 680, or donkey anti‐rabbit 680 and donkey anti‐mouse 790, Invitrogen). After washing, proteins were visualized and quantified using the Odyssey CLx and Image Studio software (LI‐COR Biosciences, USA). Protein levels were normalized to the brightest signal within each membrane, followed by normalization to β‐actin, and presented as relative to baseline (biopsies) or vehicle (Caco‐2 cells).

### 2.8 Immunofluorescence and Immunocytochemistry

Formalin‐fixed paraffin‐embedded biopsy sections were deparaffinized in xylene and rehydrated through a graded ethanol series. Heat‐induced antigen retrieval was performed by microwaving sections in citrate buffer (10 mM, pH 6.0) for 10 min. After cooling, sections were rinsed in PBS, permeabilized with 0.1% Triton X‐100 in PBS for 5 min and blocked using Background Sniper (Biocare medical, USA). Sections were incubated overnight at 4°C with primary antibodies against tricellulin (rabbit; 1:100) and ZO‐1 (goat; 1:100), diluted in glycine buffer (0,3 M glycine in PBS‐Tween 0,1%) containing 3% BSA (Sigma‐Aldrich). Following washing, sections were incubated for 1 h at RT in the dark with appropriate fluorescent secondary antibodies (donkey anti‐goat 488 and donkey anti‐rabbit 594; 1:500; Invitrogen). Nuclei were counterstained with one NucBlue Fixed cell stain (Invitrogen) for 10 min, and sections were mounted using ProLong Glass Antifade Mounant (Invitrogen).

Fixed cells were permeabilized with 0,1% Titon X‐100 in PBS for 5 min, blocked with 5% BSA in PBS for 1 h at RT, and incubated overnight at 4°C with primary antibodies against tricellulin (rabbit; 1:100) and occludin (mouse; 1:100), diluted in blocking buffer supplemented with 0,1% Triton X‐100. Following washing, cells were incubated for 1 h at RT in the dark with appropriate secondary antibodies (donkey anti‐rabbit 488 and donkey anti‐mouse 568; 1:500, Invitrogen). Nuclei were counterstained as described for biopsies and coverslips were mounted upside‐down on microscope slide with mounting medium.

### 2.9 Confocal Microscopy and Image Analysis

Stained biopsy sections and Caco‐2 cell monolayers were imaged using Stellaris 5 (Leica, Germany) confocal laser scanning microscope equipped with a 63×, 1.4 NA oil immersion objective (1.52 refractive index). Image acquisition settings (laser power, gain, offset settings) were kept constant across all samples. To quantify tricellulin enrichment at tTJ, a preliminary cell segmentation step was performed to identify tricellular vertices.

Images processing workflow was as follows: For biopsy sections, 4–6 z‐stacks from separate fields of view corresponding to crypt epithelium were acquired for each patient's slide, before and after treatment with ReFerm® or placebo. Maximum intensity orthogonal projections of 2–3 z‐planes encompassing the apical junctional belt were processed with application of TopHat and Subtract Background filter in ImageJ and analyzed with CellProfiler. Briefly, ZO‐1 channel was smoothed and enhanced using a tubeness filter, followed by thresholding with Otsu's algorithm. Tricellular vertices were identified as branch points of the corresponding skeletonized image. For cell monolayers, three z‐stacks from separate fields of view for each coverslip were acquired. Maximum intensity orthogonal projections of 2–3 z‐planes encompassing the apical junctional belt were processed with application of TopHat and Subtract Background filter in ImageJ. Cell segmentation was performed using the Tissue Analyzer in ImageJ by applying a watershed algorithm to the occludin channel. TJ and vertices masks were exported and further analyzed with CellProfiler. In CellProfiler, a region of interest (ROI) was applied to exclude out‐of‐focus areas and tricellulin intensity was measured within a circular region centered at tTJ and at bTJ, 2 μm from vertices. Tricellulin enrichment at tTJ was calculated as the ratio between mean fluorescence intensity at tTJ and bTJ, as previously described [66]. In addition, tricellular vertices were classified as tricellulin‐positive or ‐negative using a per image threshold defined as the median tricellulin intensity at bTJs plus two times the median absolute deviation (MAD). The percentage of tricellulin‐positive vertices was calculated and reported as the fraction of vertices exhibiting enriched tricellulin localization. Analysis pipelines used in study are available upon request.

### 2.10 ELISA And EIA

Plasma levels of TNF and VIP were analyzed in duplicates using an ultrasensitive TNF ELISA kit (Invitrogen, Thermo Fisher Scientific) and a VIP EIA kit (Phoenix Pharmaceuticals, Germany), according to manufacturer's instructions and as previously described [28, 29].

### 2.11 Statistics

Statistical analysis was performed using GraphPad Prism software version 10.0.2 (Boston, MA, USA). Data were screened for outliers using ROUT test with the Q set at a 1% cutoff and checked for normality using the Shapiro–Wilks test. For normally distributed data, comparisons between groups were performed using analysis of variance (ANOVA) or Student's *t*‐test, and results were presented as the mean ± the standard error of the mean (SEM). For non‐parametric data, comparisons between groups were performed using Wilcoxon signed‐rank test, and results were presented as the median with interquartile range. For correlation analysis, Spearman's non‐parametric correlation coefficient was calculated, and a linear regression analysis was performed between change in TJ protein expression and symptoms improvement, evaluated by the difference in IBS‐SSS before and after the intervention.

## 3 Results

### 3.1 Questionnaires

Results from the questionnaire outcomes are shown in Table 1. Marked improvement was defined as a decrease of at least 50 points in the IBS‐SSS.

### 3.2 Decreased Permeability in Biopsies Exposed to ReFerm®

**FIGURE 2:**
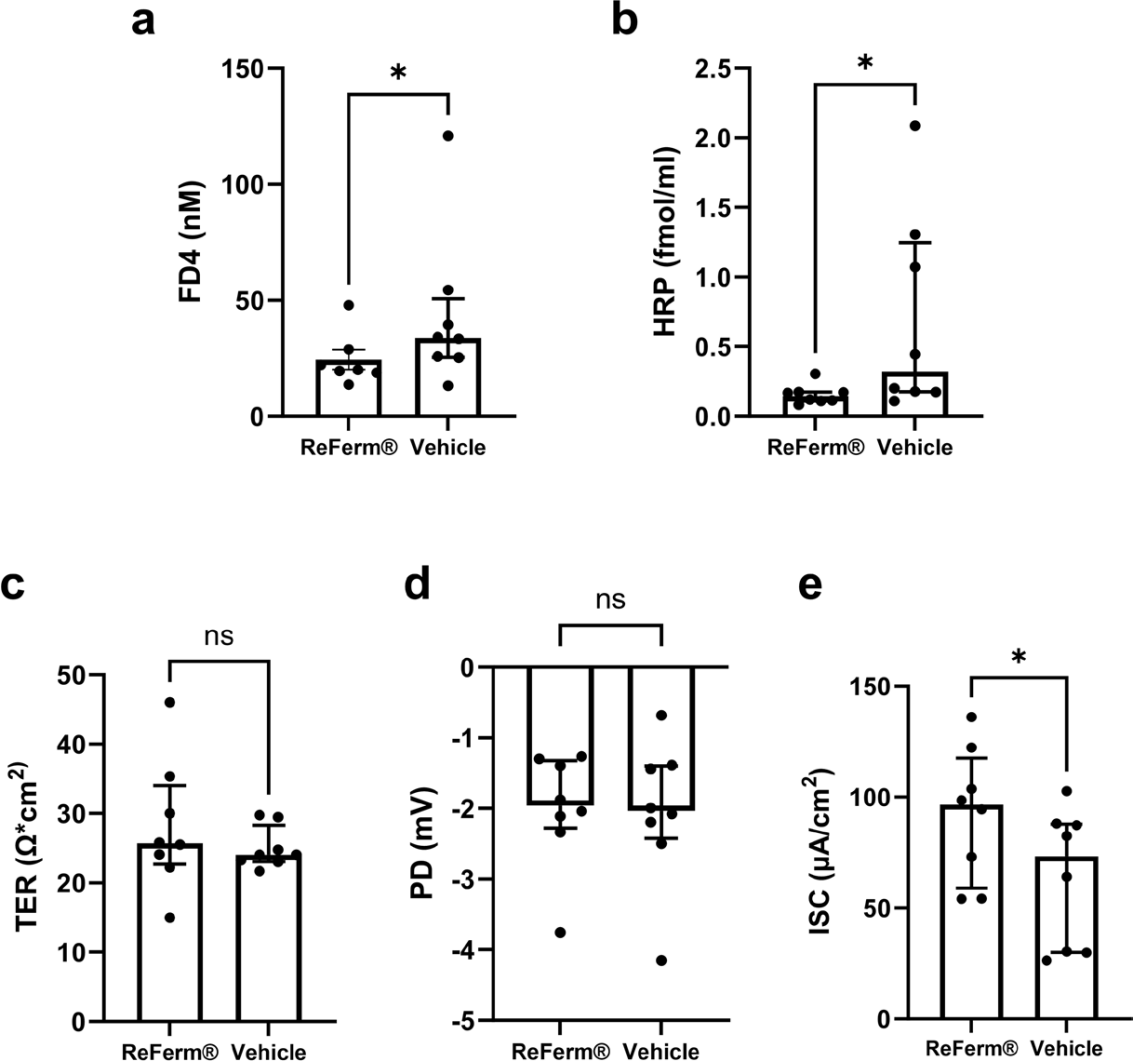
Effects of the fermented oat gruel composition ReFerm® on intestinal barrier function. Colonic biopsies from 8 patients with irritable bowel syndrome (IBS) were mounted in Ussing chambers, added ReFerm® or vehicle (Krebs buffer only) to the mucosal sides, and permeability was measured over time. (a) Paracellular permeability to fluorescein isothiocyanate (FITC)‐dextran 4 kDa (FD4) was significantly reduced by ReFerm® compared to vehicle. (b) Transcellular passage to horseradish peroxidase (HRP) was significantly reduced by ReFerm® compared to vehicle. There was no significant effect of ReFerm® on the transepithelial electrical resistance (TER) (c) or potential difference (PD) (d); however, the short‐circuit current (Isc) was significantly increased by ReFerm® compared to vehicle (e). Graphs show the 30–90 min passage, and TER, PD and Isc at 90 min. Data are presented as scatter plots representing individual patients, with bars indicating median (interquartile range), *p < 0.05, ns = non‐significant. Statistical analysis was performed using Wilcoxon signed‐rank test.

Biopsies treated directly with ReFerm® (diluted 1:20) in the Ussing chambers demonstrated a lower paracellular permeability compared with the vehicle (Figure 2a). The same pattern was observed for transcellular passage (Figure 2b). ReFerm® diluted 1:100 had no significant effect on permeability compared with vehicle (FITC‐dextran: ReFerm® 1:100; 41.3 ± 15,1 nM and Vehicle 43.3 ± 11.9 nM; HRP: ReFerm® 1:100 0.33 ± 0.06 fmol/ml and vehicle 0.70 ± 0.2 fmol/ml).

There were no significant effects on TER (Figure 2c) or PD (Figure 2d) by exposure to ReFerm®, while Isc was significantly increased after ReFerm® stimulation compared with vehicle (Figure 2e).

### 3.3 Increased Expression of Tricellulin and Subcellular Enrichment at tTJ in Biopsies From IBS Patients Treated With ReFerm®

**FIGURE 3:**
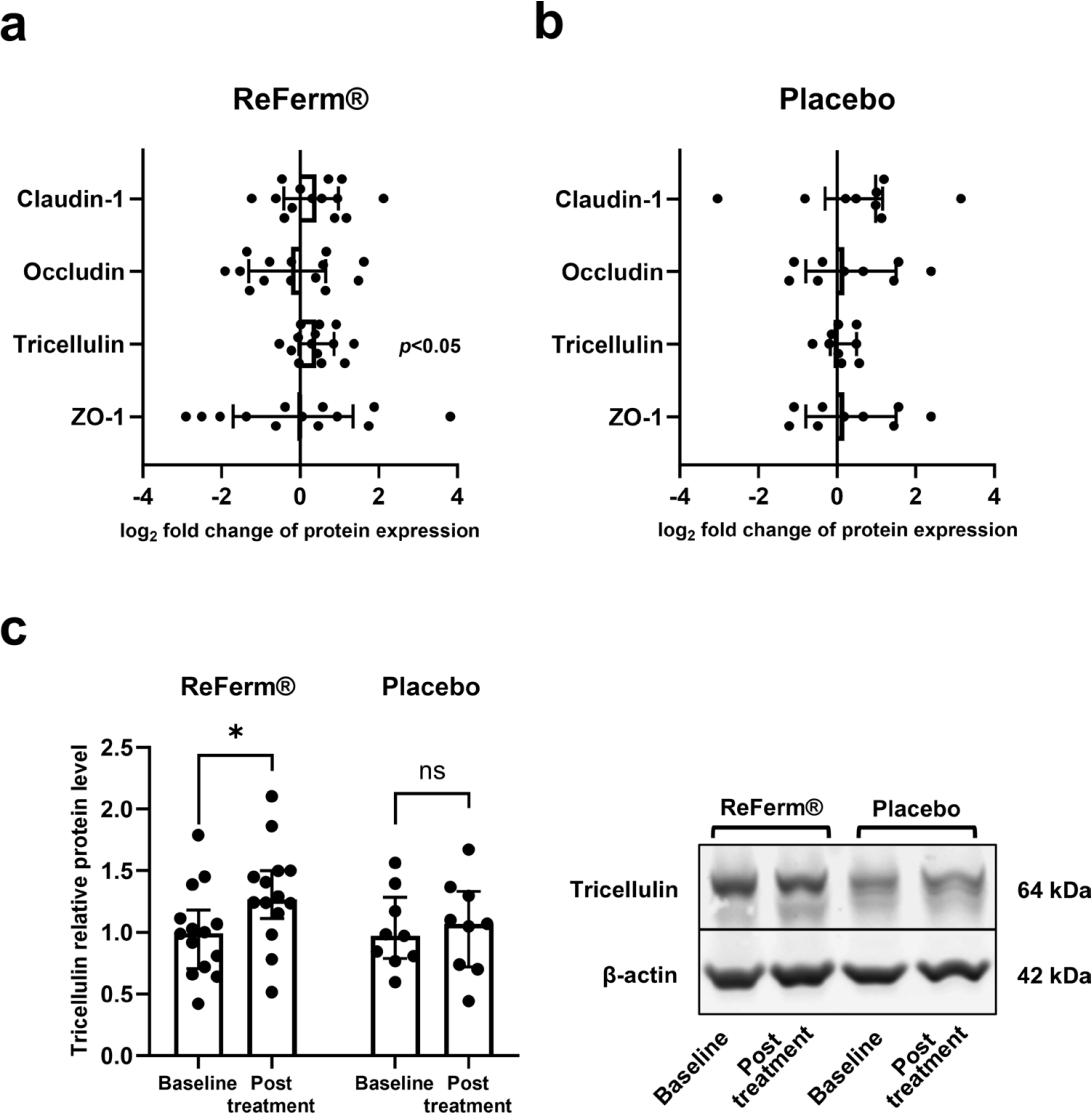
Relative expression (log2 fold change) of tight junction (TJ) proteins claudin‐1, occludin, tricellulin, and ZO‐1 measured by Western blotting in colonic biopsies of patients with irritable bowel syndrome (IBS) before and after enema treatment with ReFerm® (*n* = 14) or placebo (*n* = 9). Band intensities were normalized to β‐Actin as a loading control and expressed as log2 fold change relative to the baseline condition (before treatment). (a) ReFerm® significantly increased the tricellulin expression. (b) Placebo had no effect on TJ protein expressions. (c) Scatter plots showing the significant effect of ReFerm® on tricellulin expressions (presented as relative to baseline), while placebo had no effect. Photograph shows representative Western blot membranes for tricellulin with β‐Actin as a loading control. Data are presented as scatter plots representing individual patients, with bars indicating median (interquartile range). Statistical analysis was performed using Wilcoxon signed‐rank test on normalized band intensities, *p < 0.05, ns = non‐significant.

A 14‐day treatment with ReFerm® administered by enema increased tricellulin protein expression, *p* < 0.05, measured by Western blotting in colonic biopsies from patients with IBS, whereas no change was observed in the placebo group (Figure 3a–c). The expression of claudin‐1, ZO‐1, and occludin remained unchanged in both groups (Figure 3a,b). There was no difference in tricellulin expression between IBS subgroups, neither in ReFerm® nor in placebo (data not shown).

**FIGURE 4:**
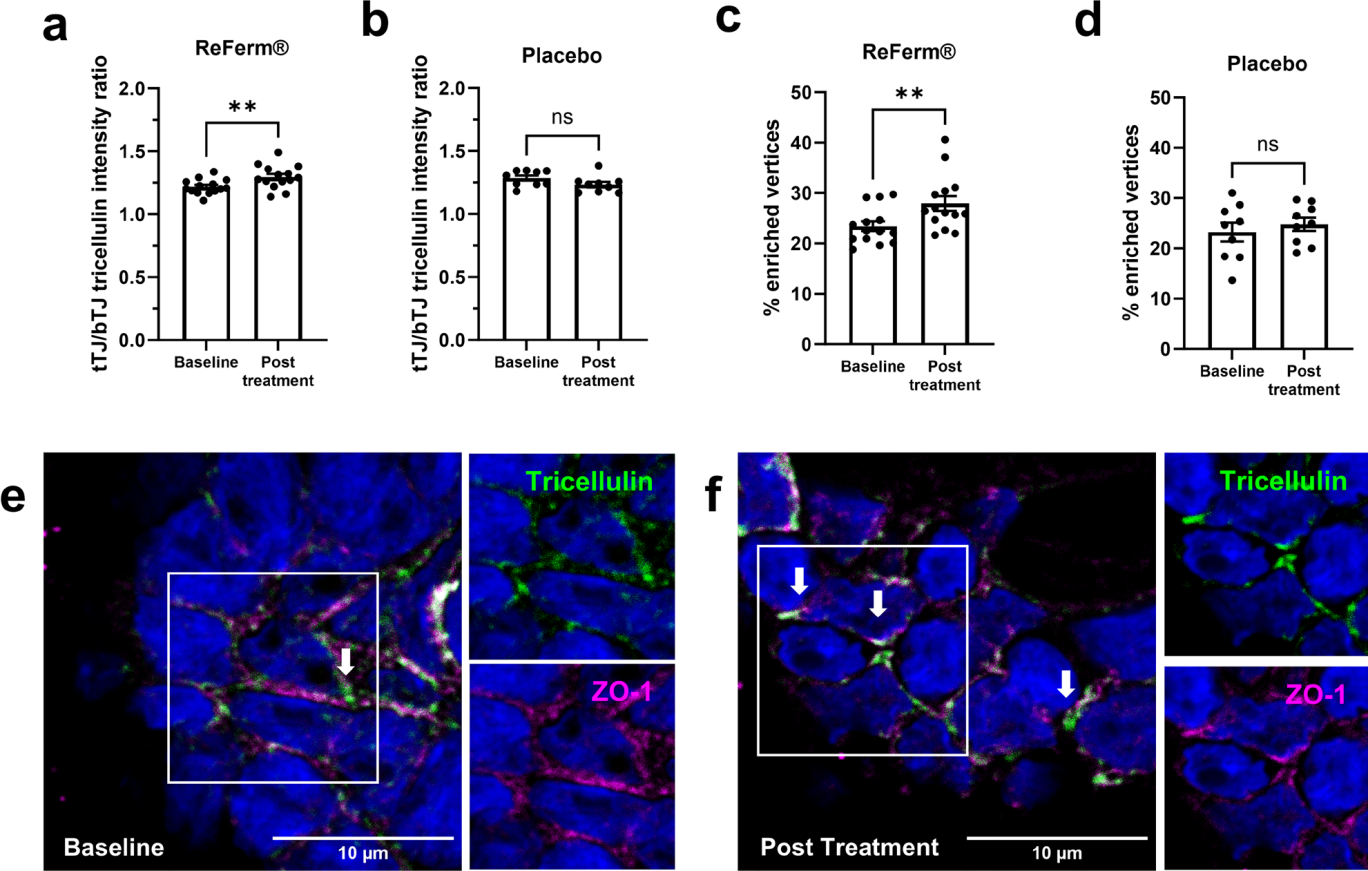
The effect of ReFerm® and placebo on tricellulin expression and tricellulin enrichment in colonic biopsies from patients with irritable bowel syndrome (IBS). Sections were stained with antibodies against tricellulin (green) and ZO‐1 (pink). Tricellulin intensity was quantified at tricellular junctions (tTJ) and normalized to the intensity at bicellular junctions (bTJ) measured 2 μm from the tTJ. (a) There was a significantly higher ratio between tTJ and bTJ after ReFerm® treatment, indicating increased tricellulin enrichment, while there was no effect by placebo (b). Tricellular vertices were classified as tricellulin‐positive or ‐negative using a threshold defined as the median tricellulin intensity at bTJs plus two times the median absolute deviation. (c) There was an increased fraction of vertices enriched in tricellulin after ReFerm® treatment, while no effect was seen by placebo (d). Representative confocal images before ReFerm® treatment at baseline (e), and post treatment (f). Arrows point to tTJ enriched in tricellulin. Staining of ZO‐1 indicates the bTJ. Results are shown as scatter plots representing individual patients, with bars indicating mean ± SEM, **p < 0.01, ns = non‐significant. Statistical analysis was performed using paired Student's T test.

Confocal microscopy revealed an increase in tricellulin intensity at tTJ, normalized to the intensity measured at the bTJ 2 μm from tricellular vertices in biopsies of patients treated with ReFerm®, *p* < 0.01, compared with baseline (Figure 4a), while no difference was observed in the placebo group (Figure 4b). The percentage of enriched vertices, determined using a per‐image threshold defined as the median tricellulin intensity at bTJs plus two times the MAD, was also increased following ReFerm® treatment, *p* < 0.01 (Figure 4c), while remaining unchanged in the placebo group (Figure 4d).

Subgroup analysis revealed that the difference in tricellulin was primarily driven by patients with IBS‐M. This applied to both intensity at tTJ (mean ± SEM: IBS‐M: baseline: 1.21 ± 0.01 and post treatment:1.31 ± 0.03; IBS‐D: baseline: 1.24 ± 0.05 and post treatment: 1.25 ± 0.05) and tricellulin enrichment (IBS‐M: baseline: 22,6 ± 1,17 and post treatment: 29.0 ± 1.8; IBS‐D: baseline: 25.6 ± 1.4 and post treatment: 25.2 ± 1.9). In contrast, no differences between IBS subgroups were detected in the placebo group (data not shown). Representative images are shown in Figure 4e,f.

### 3.4 ReFerm® Stimulation in Caco‐2 Cells Improves Subcellular Tricellulin Enrichment at tTJs

**FIGURE 5:**
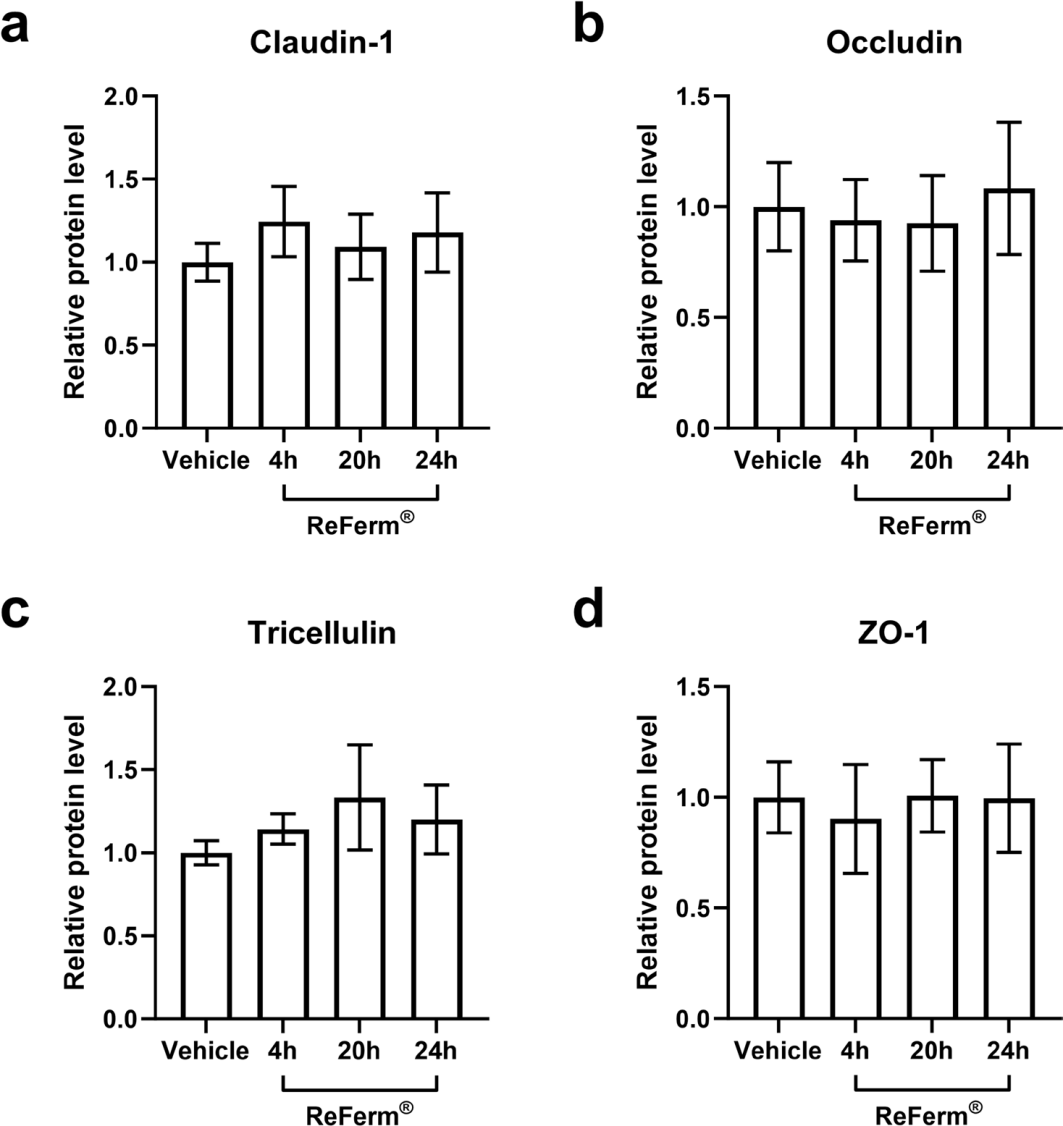
Effects of ReFerm® on tight junction (TJ) protein levels in Caco‐2 cells measured by Western blotting. Band intensities were normalized to β‐Actin in the corresponding lane and expressed relative to the mean value of the cells added cell culture media only (vehicle). (a‐d) There was no significant effect on any of the TJ investigated at any time point. Data are based on four independent experiments. Statistical analysis was performed using one‐way ANOVA followed by Dunnett's multiple comparisons test.

Densitometric analysis of Western blots showed no significant changes in the expression levels of TJ proteins claudin‐1, occludin, tricellulin and ZO‐1 in Caco‐2 cells at any of the analyzed time points after treatment with ReFerm® 1:20 compared to un‐treated cells (vehicle) (Figure 5a–d). The same pattern was seen for ReFerm® 1:100 with no significant difference for any of the TJ investigated (data not shown).

**FIGURE 6:**
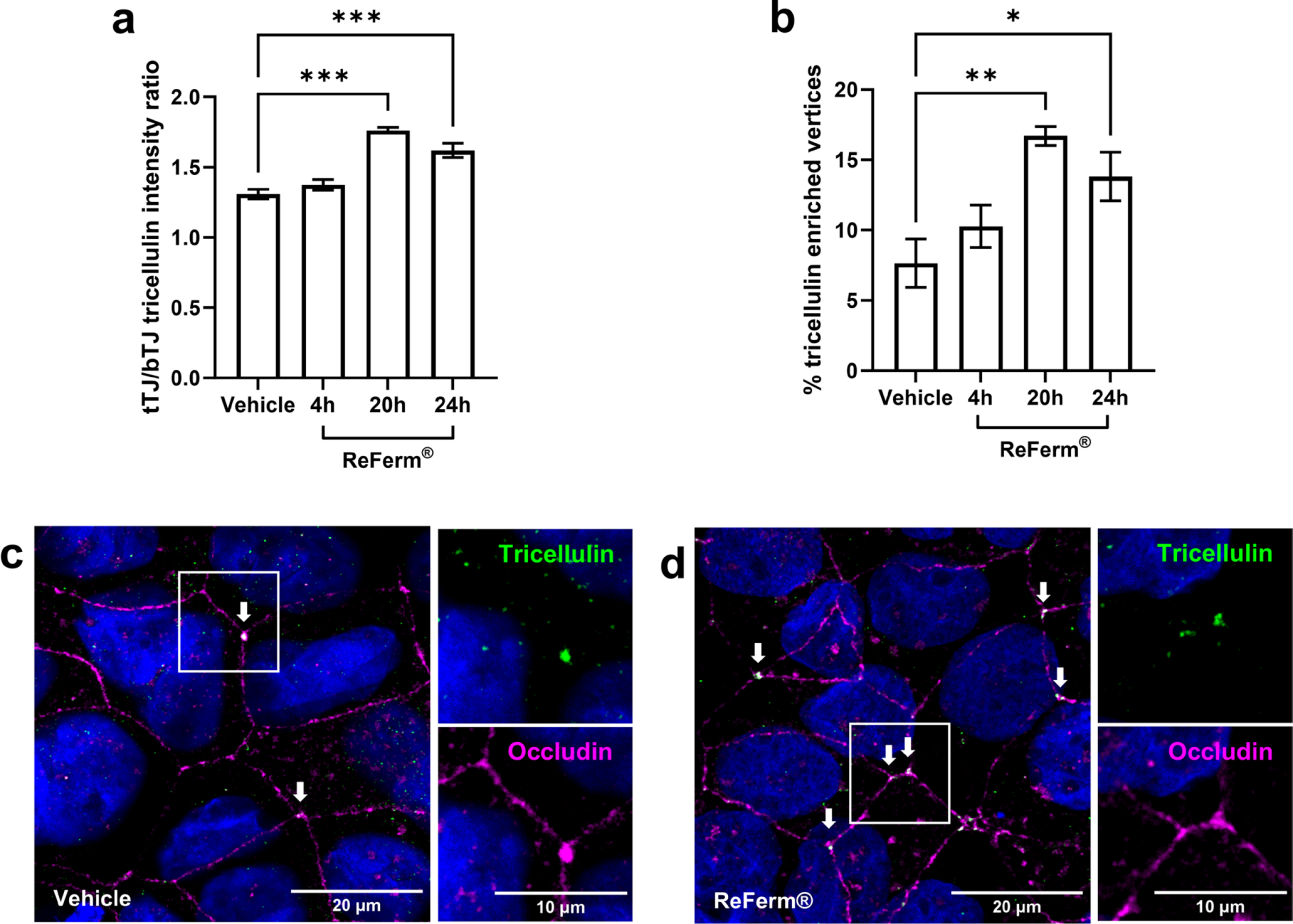
The effect of ReFerm® on tricellulin expression and tricellulin enrichment in Caco‐2 cells. Cells were stained with antibodies against tricellulin (green) and occludin (pink). (a) Tricellulin intensity was quantified at tricellular junctions (tTJ) and normalized to the intensity at bicellular junctions (bTJ) measured 2 μm from the tTJ. A higher ratio indicates increased tricellulin enrichment at tTJs after 20 h (h) and 24 h of ReFerm® treatment compared to vehicle (un‐treated cells). Maximum enrichment was reached at 20 h and remained significant at 24 h. (b) Tricellular vertices were classified as tricellulin‐positive or ‐negative using a threshold defined as the median tricellulin intensity at bTJs plus two times the median absolute deviation. A higher percentage indicates increased fraction of vertices enriched in tricellulin after 20 h and 24 h of ReFerm® treatment. Maximum percentage of enriched vertices was reached at 20 h and remained significant at 24 h. Representative confocal images of tricellulin in (c) Caco‐2 cells treated with vehicle (d) Caco‐2 cells exposed to ReFerm®. Arrows point to tTJs enriched in tricellulin. Data are presented as the mean ± SEM of three independent experiments. Statistical analysis was performed using one‐way ANOVA followed by Dunnett's multiple comparisons test.

Confocal microscopy of Caco‐2 cell monolayers treated with ReFerm® 1:20 revealed a significant increase in tricellulin enrichment at tTJ compared to vehicle (Figure 6a). The enrichment was evaluated as the tTJ/bTJ intensity ratio and as the percentage of tricellular vertices with enriched tricellulin content as in Figure 6b. The effect was time‐dependent, with the highest values observed at 20 h, while remaining significantly elevated at 24 h. Representative images are shown in Figure 6c,d. Caco‐2 cell monolayers treated with ReFerm® 1:100 had no significant effect on tricellulin enrichment compared to vehicle (data not shown).

### 3.5 Plasma VIP Levels Remained Unchanged Following ReFerm® Treatment, While TNF Levels Indicate a Protective Effect

**FIGURE 7:**
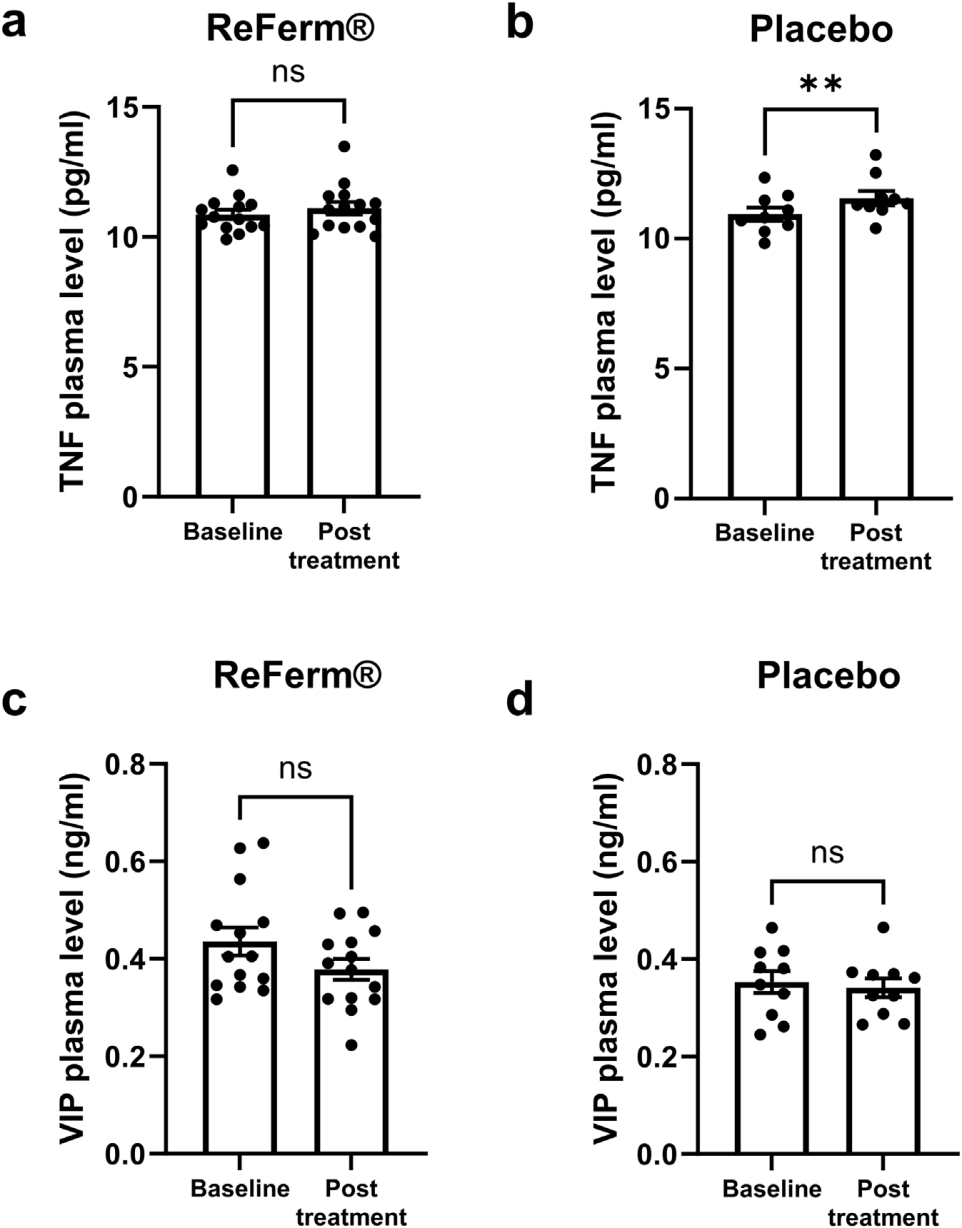
Levels of TNF and vasoactive intestinal polypeptide (VIP) in plasma of patients with irritable bowel syndrome. Concentrations were measured at baseline and after enema treatment with ReFerm® or placebo for 14 days. Levels of TNF were unchanged in the ReFerm® group (a) while significantly increased in the placebo group (b). There was no significant difference in plasma levels of VIP before and after treatment with ReFerm® (c) or placebo (d). Results are shown as scatter plots representing individual patients, with bars indicating mean ± SEM. Statistical analysis was performed using paired Student's *t* test. ns = non‐significant.

Plasma concentrations of VIP and TNF were measured at baseline and after a 14‐day treatment with ReFerm® or placebo administered by enema. TNF levels were significantly increased in the placebo group while unchanged in patients treated with ReFerm® (Figure 7a,b), suggesting a protective role of ReFerm®. VIP levels remained unchanged in both groups (Figure 7c,d).

### 3.6 Relation Between Gut Findings and Clinical Data

No associations were found between age, BMI, or disease duration in IBS patients and any of the measured study parameters (data not shown). Likewise, no significant correlations were observed between questionnaire outcomes and measures of barrier function and TJ, either in pooled analyses including all patients or in subgroup analyses (data not shown).

## 4 Discussion

This study shows that the dietary product ReFerm®, manufactured by fermenting an oat composition using *L. plantarum* DSM 9843, significantly improves intestinal barrier function when applied to colonic biopsies from patients with IBS via mechanisms including the TJ protein tricellulin. We have previously reported that ReFerm® strengthens the intestinal barrier by reducing paracellular permeability and increasing TER [22], as well as by reducing colonic mast cell degranulation [23] in patients who received ReFerm® enema for 14 days. In the present study, we can confirm a decreased paracellular permeability by ReFerm®. Furthermore, we demonstrate a reduction in transcellular passage, indicating a more comprehensive improvement in epithelial barrier function. This effect was not observed in our previous study using biopsies from patients receiving ReFerm® as an enema; however, when ReFerm® was applied to Caco‐2 cells, both paracellular and transcellular permeabilities were reduced [22], supporting the findings of the present study. In contrast to our previous in vivo findings [22], no significant effect on TER was observed in the present ex vivo model, likely reflecting differences in experimental conditions. While earlier results were obtained after repeated in vivo exposure over 14 days, the current experiments assess acute direct effects on biopsies, suggesting that changes in TER may require longer exposure time and that permeability endpoints may be more sensitive in detecting early barrier responses. Given that HRP flux was also reduced when ReFerm® was applied directly to biopsies in Ussing chambers, future studies should additionally explore transcellular pathways to better define the molecular mechanisms of ReFerm® underlying these effects. Variability in HRP flux is consistent with previous observations [22, 28] that transcellular permeability shows greater inter‐individual variability compared with paracellular pathways.

A large body of evidence supports a role for increased intestinal permeability as a key contributor to IBS pathophysiology [2, 3, 4]. Translocation of luminal antigens can activate mucosal immune cells, promoting the release of mediators that sensitize enteric nerve terminals, ultimately leading to visceral hypersensitivity and abdominal pain. TJ are main regulators of this process, controlling paracellular permeability to luminal macromolecules. In the present study we showed an increased Isc by ReFerm®, which in some contexts can be associated with barrier disturbance. However, Isc primarily reflects net ion transport rather than barrier integrity per se [30], and potentially represents a mucosal defense response rather than epithelial injury, supported by unchanged TER and reduced permeability. Indeed, Ussing chamber studies distinguish between paracellular permeability and electrophysiological parameters such as Isc and TER. In the present study, the increase in Isc is therefore more likely to reflect enhanced epithelial ion secretion rather than barrier disruption. Increased Isc is commonly linked to active chloride secretion and subsequent fluid movement into the lumen [31, 32]. Such secretory responses are considered important components of mucosal defense, facilitating the clearance of luminal microbes and toxins [33]. In this context, the observed increase in Isc by ReFerm® may represent a protective response, while barrier integrity appears preserved as supported by the unchanged TER and reduced paracellular permeability observed in parallel. From a clinical perspective, these effects may be particularly relevant in IBS‐C, where increased luminal hydration and secretion could be beneficial, while in IBS‐D, the implications are less clear.

Two distinct paracellular pathways have been defined in the intestinal epithelium; the pore pathway, which enables size‐selective ion transport, and the leak pathway, which permits macromolecular flux [34]. While the expression of subsets of pore‐forming claudins primarily regulates the pore pathway, tTJs are considered major sites for the leak pathway. Studies have generally reported a reduced expression of either claudin‐1, ZO‐1, or occludin in IBS patients compared to HC [3, 4]. We did not observe any change in the expression of these proteins in colonic biopsies from IBS patients following 14 days of ReFerm® enema treatment. However, by Western blotting, we found a modest but statistically significant increase in tricellulin protein expression after ReFerm® treatment, while no change in the placebo group. This finding is in line with the reduction observed in paracellular permeability after ReFerm® treatment, and in combination with the absence of changes in TER, it strongly supports a tTJ remodeling. As other authors reported [35, 36], permeability to FITC‐dextran 4 kDa can be modulated through the overexpression of tricellulin, the key component of tTJ, without affecting TER. Whether such modest changes in tricellulin protein level are sufficient to have an impact on paracellular permeability may be debated. However, the subcellular localization of tricellulin is likely to be more relevant for paracellular leakage than overall protein expression. In this regard, Awad et al. [13] recently analyzed tricellulin localization in a subgroup of patients with IBS‐M using confocal and super‐resolution microscopy, reporting a dislocation from tTJ to bTJ. To assess the effect of ReFerm® on tricellulin subcellular localization, we performed confocal imaging and quantified the protein enrichment at the tTJ by comparing the percentage of enriched vertices and fluorescent intensity normalized to bTJ in colonic biopsy sections of patients before and after ReFerm® or placebo treatment. We observed a significant increase in tricellulin enrichment at tTJ in the ReFerm® group after 14 days of treatment, while no effect was detected in the placebo group. Notably, while the overall effect remained significant, subgroup analysis revealed that this difference was driven primarily by patients with IBS‐M. However, this finding should be interpreted with caution considering the low number of patients after sub‐dividing them, especially in the IBS‐D group (IBS‐M; *n* = 10, IBS‐D; *n* = 4). Importantly, a similar increase in tricellulin enrichment at tTJs was also observed in Caco‐2 cells, supporting the consistency of this effect across experimental models. The expression and organization of TJ proteins in epithelial barriers are finely regulated through immune‐mediated mechanisms [37] and tricellulin expression is, for example, downregulated in the colon of patients with ulcerative colitis via type 2 cytokines signaling, including IL‐13 [38] and restored during disease remission [39]. Alterations in IL‐13 signaling have been reported in IBS as well [40]. In our settings, ReFerm® exerted a protective effect on low‐grade inflammation by preventing the increase in plasma TNF levels observed in the placebo group. However, the observed increase in TNF in the placebo group was modest and is unlikely to be clinically significant. We do not interpret this as evidence that repeated rectal enemas impair barrier function, but rather as reflecting a mild inflammatory response to the intervention itself. In this context, and consistent with our previous findings of reduced mast cell degranulation following ReFerm® treatment, the absence of a TNF increase in the active group may indicate reduced mucosal immune activation and suggest a protective or stabilizing effect of ReFerm®. Nevertheless, this interpretation remains speculative, and the present study does not allow for mechanistic conclusions. In addition, we previously reported [23] that ReFerm® treatment significantly reduced mast cell degranulation. Mast cells can compromise intestinal barrier integrity through the release of TNF, which disrupts TJ via myosin light‐chain kinase activation and downregulation of proteins such as ZO‐1 and occludin, resulting in increased epithelial permeability [41]. Taken together, these findings raise the possibility that the effects of ReFerm® on tricellulin expression and localization may be partly mediated by changes in the local pro‐inflammatory milieu, although this mechanism was not directly assessed in the present study.

*L. plantarum* DSM 9843 has shown to be beneficial in a variety of gastrointestinal conditions suggested by multiple mechanisms, including modulation of TJ protein expression, mucus secretion, immunomodulatory activity, and interaction with host microbiota [42]. In the present study, we show that the fermentation product from oats fermented with *L. plantarum* DSM 9843 improves intestinal barrier through a novel mechanism, namely the modulation of tricellulin expression and subcellular localization. The composition of fermentation products like ReFerm® is very complex. ReFerm® contains both oat components and many microbial metabolites produced during the fermentation. It is likely that it is not one or a very few molecules in ReFerm® that provide the biological effect. Rather, it is a relatively large plurality of molecules in ReFerm® that together provide the biological effect. This is a challenging model, but also interesting because it leaves the traditional box that says one molecule, one effect, and makes room for new mechanisms of action within, for example, postbiotics.

Bertiaux–Vandael et al. [4] demonstrated that occludin and claudin‐1 levels in patients with IBS correlate with both longer symptom duration and greater abdominal pain intensity. Recently, Awad et al. [13] showed that IBS‐M patients exhibit tricellulin delocalization together with increased epithelial permeability, changes that plausibly contribute to core IBS symptoms through barrier dysfunction and immune activation. In the present study, tricellulin expression and enrichment were not associated with clinical symptoms in either analyses of the full cohort or when stratified by IBS subgroups, although the relatively small cohort may have limited the ability to detect such associations.

The present work has some limitations that should be acknowledged. First, this study was exploratory in nature, and the study was not powered for subgroup analyses, reducing the statistical power. These results should therefore be interpreted in this perspective. Furthermore, placebo responses are known to be strong in IBS clinical trials [43]. While the regular patient–clinician contact in our study could potentially have contributed to such an effect, we did not observe any notable clinical improvement in IBS symptoms following any of the interventions. However, our study was a proof‐of‐concept investigation designed to explore the product's mechanism of action rather than its clinical effect. Another limitation might be that the short intervention period in our study may have limited observable changes in permeability; treatments (probiotic) are generally recommended for 4–12 weeks, which is longer than the duration used here. Except from larger cohorts, future studies should also include oral administration to assess the product's clinical efficacy in IBS patients. An additional limitation is the lack of quantitative and detailed characterization of the product composition, including individual components such as SCFAs and β‐glucans, which limits identification of the specific active molecules and their mechanisms of action. As suggested above, the observed effects are likely mediated by a combination of multiple components acting in concert, rather than by a single molecule. Importantly, the experimental design, based on rectal administration or direct application to biopsies, ensured high local concentrations at the colonic mucosa, which does not reflect oral intake where dilution, digestion, and metabolic transformation would occur prior to reaching the colon. This complicates direct translation of the findings to expected luminal concentrations after ingestion. While a more detailed compositional and mechanistic understanding, including the contribution of specific molecular fractions, would be highly informative, such characterization would require substantial effort, including identification of numerous components and functional testing of individual molecules and their combinations. These aspects remain important areas for future research.

In conclusion, ReFerm® improves both paracellular and transcellular permeability when applied directly to biopsies of IBS patients. The improvement in paracellular permeability appears to occur through a mechanism involving the redistribution of tricellulin at tTJ. Larger studies are warranted to confirm these findings and to further elucidate the regulation of both paracellular and transcellular pathways. In addition, studies assessing the effects of the product administered as a beverage on clinical symptoms are needed.

## Author Contributions

V.A.: Performed immunofluorescence staining, microscopy and quantification of TJ in patients with IBS and in Caco‐2 cells; performed in vitro experiments and Western blotting; involved in analysis and interpretation of data; involved in the concept and design of the study; drafted the manuscript; revised the manuscript after revision from co‐authors. O. Biskou: Performed in vitro experiments; participated in Ussing chamber experiments; involved in the concept and design of the study; revised the manuscript critically for important intellectual content. M.E.W.: Performed Ussing chamber experiments and Western blotting; revised the manuscript critically for important intellectual content. H.I.: Involved in the concept and design of the study; revised the manuscript critically for important intellectual content. O. Bednarska: Included IBS patients and performed sigmoidoscopy; compiled the patients' characteristics; involved in the concept and design of the study; funded parts of the study; revised the manuscript critically for important intellectual content. S.W.: Included IBS patients and performed sigmoidoscopy; involved in the concept and design of the study; involved in interpretation of data; revised the manuscript critically for important intellectual content. Å.V.K.: Responsible for the final concept and design of the study; involved in analysis of data; responsible for the interpretation of data and the final presentation of it; funded parts of the study; drafted parts of the manuscript and revised the manuscript critically for important intellectual content; responsible for the final version of the manuscript.

## Funding

This work was supported by grants from the “P. Håkanssons Stiftelse, Eslöv, Sweden” (OBe) “Medical Infection and Inflammation Center‐MIIC” (ÅVK), “Grants, Region Östergötland” (LIO‐938303 OBe and RÖ‐960493, RÖ‐969343 ÅVK), “Bengt Ihre Foundation” (OBe), “Ruth and Richard Julin Foundation 2022‐00270” (ÅVK), “Stiftelsen Apotekare Hedbergs fond för medicinsk forskning” (ÅVK).

## Conflicts of Interest

H.I. was employed by Nordic Rebalance, which provided partial funding for this study; however, this had no influence on the study's content. The remaining authors state that the research was carried out without any commercial or financial relationships that could be interpreted as a potential conflicts of interest.

## Data Availability

The data that support the findings of this study are available on request from the corresponding author. The data are not publicly available due to privacy or ethical restrictions.
